# Early maternal weight gain as a risk factor for SGA in pregnancies with hyperemesis gravidarum: a 15-year hospital cohort study

**DOI:** 10.1186/s12884-020-02947-3

**Published:** 2020-04-28

**Authors:** Tale Meinich, Jone Trovik

**Affiliations:** 1 Department of Clinical Science, University of Bergen, Jonas Lies vei 72, 5053 Bergen, Norway; 2 Department of Obstetrics and Gynaecology, Haukeland University Hospital, Jonas Lies vei 72, 5053 Bergen, Norway

**Keywords:** Body mass index (BMI), Gestational weight gain, Hyperemesis gravidarum, Small for gestational age (SGA)

## Abstract

**Background:**

Inadequate maternal weight gain increases the risk of small-for-gestational age (SGA) infants. Women with hyperemesis gravidarum (HG) are at risk of significant early pregnancy weight loss and insufficient total pregnancy weight gain. Recent studies have implied that weight gain during the first half of pregnancy is more crucial to pregnancy outcome than total weight gain.

The aim of this study was to investigate whether not regaining prepregnancy weight by 13–18 weeks of gestation contributed to not reaching minimum body mass index (BMI)-specific total pregnancy weight gain and influenced the risk of SGA outcome in HG pregnancies.

**Methods:**

In this retrospective 15-year cohort (2002–2016) of women hospitalized due to hyperemesis gravidarum, we reviewed individual patient hospital files and corresponding outpatient maternity records to collect prepregnancy BMI and weight, pregnancy weight gain (spanning 3-week intervals), delivery weight and foetal outcomes. BMI and total pregnancy weight gain goals were categorized according to the Institute of Medicine (IOM) 2009 guidelines: BMI < 18,5 kg/m^2^: 12.5–18 kg, 18.5–24.9 kg/m^2^: 11.5–16 kg, 25–29.9 kg/m^2^: 7–11.5 kg and > 30 kg/m^2^: 5–9 kg. Birth weight was categorized as SGA if less than the 10th percentile of sex- and gestational length-specific Norwegian neonatal weight charts. Nonparametric tests were used to compare weight categories, and logistic regression was used to predict the odds ratio (OR) of inadequate total pregnancy weight gain or SGA delivery.

**Results:**

Out of 892 women hospitalized for HG during 2002–2016, 784 had a pregnancy lasting > 24 weeks, of which 746 were singleton pregnancies with follow-up until delivery. Among these women, 42 were classified as underweight, 514 as normal weight, 230 as overweight and 102 as obese before pregnancy. Not regaining prepregnancy weight by week 13–18 was an independent predictor of inadequate total gestational weight gain with an OR of 7.05 (95% CI 4.24–11.71) and an independent predictor for SGA outcome with an OR of 2.66 (95% CI 1.11–6.34), even when adjusted for total pregnancy weight gain, prepregnancy BMI, parity, age and smoking status.

**Conclusion:**

Inadequate total maternal weight gain and not regaining prepregnancy weight by week 13–18 may be considered independent risk factors for delivering a baby that is small for gestational age in pregnancies with hyperemesis gravidarum. Achieving adequate weight gain during the first trimester in HG pregnancies is important for the foetal outcome, underscoring the importance of nutritional treatment during this period.

## Background

Hyperemesis gravidarum (HG) is an extreme form of nausea and vomiting in pregnancy (NVP) that affects approximately 1% of pregnant women, but its prevalence varies from 0.3–10.8% based on different population demographics [1]. The mechanisms behind NVP and HG are not fully understood. NVP is very common, affecting 70–80% of all pregnant women, typically self-limiting and associated with mostly favourable delivery and birth outcomes [1, 2]. HG most commonly is defined as persistent nausea and vomiting starting before the 20th week of pregnancy, leading to reduced general condition with dehydration, weight loss, and fluid and electrolyte disturbances; thus, HG usually requires admittance to the hospital and medical treatment [1, 3] and is associated with reduced quality of life for the woman [4] and increased risk for preterm delivery and small-for-gestational age (SGA) babies [5, 6]. Women suffering from HG usually lose substantial weight during early pregnancy and often struggle to achieve the recommended pregnancy weight gain [7]. Nausea and vomiting may lead to a nutritional intake of less than half of recommended values [8] and cause serious maternal complications such as Wernicke’s encephalopathy. Inadequate weight gain is a serious complication of HG [9], and a total weight gain < 7 kg has been linked to an increased risk of preterm and SGA delivery [5, 6]. Both low prepregnancy body mass index (BMI) [10] and inadequate total weight gain [6, 11] have independently been described as predictors for SGA birth. Additionally, maternal prepregnancy BMI is generally correlated with foetal weight [12–14], varying gestational weight gain [12] and maternal and neonatal morbidity in general [15, 16].

In 2018, a preconception cohort study including 1164 healthy pregnant women investigated whether the timing of maternal weight gain during pregnancy affected infant birth weight, concluding that maternal weight status in the first half of gestation, rather than the second half, is a determinant of infant birth weight [17]. Such knowledge, specifying the first trimester as a crucial window regarding maternal weight gain and pregnancy outcome, could be helpful in preventing SGA birth, especially in patients treated for HG during early pregnancy. The aim of this study was to investigate whether inadequate weight gain during the first trimester of HG pregnancies is an independent risk factor for inadequate total maternal weight gain during pregnancy, as well as an independent risk of SGA birth. Second, we investigated whether inadequate maternal weight gain during the first trimester, inadequate total maternal weight gain or SGA outcomes in HG pregnancies differ within maternal prepregnancy BMI categories.

## Methods

Women hospitalized due to HG at the Department of Obstetrics and Gynecology, Haukeland University Hospital, Bergen, Norway, during 2002–2016 were included in this retrospective cohort study. Hyperemesis gravidarum was defined as nausea and vomiting presenting before the 20th week of pregnancy, leading to reduced general well-being and at least one documented metabolic complication such as weight loss, dehydration, serum electrolyte disturbances or ketonuria not caused by other specific medical disorders. Individual patient files and outpatient maternity records were reviewed, and data regarding maternal prepregnancy weight and height (self-reported on antenatal pregnancy forms), weight measured at admissions and at outpatient antenatal appointments, maternal weight at delivery, and the corresponding birth weight and sex of the child were retrieved. Patients for which data on prepregnancy BMI, maternal weight at delivery, birth weight and gestational age at delivery > 24 weeks were available were included in calculations investigating maternal weight development. Only singleton pregnancies were included in calculations involving foetal outcome.

**Fig. 1:**
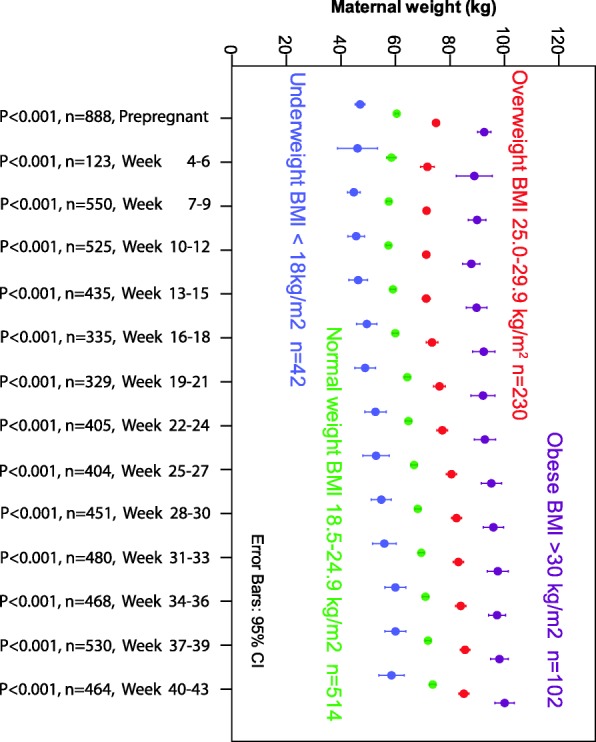
Weight gain patterns for 888 women hospitalized for hyperemesis gravidarum according to their prepregnancy body mass index (BMI) categories. Weight measured during 3-weeks interval during pregnancy until delivery. Comparisons by Kruskal-Wallis test

**Fig. 2:**
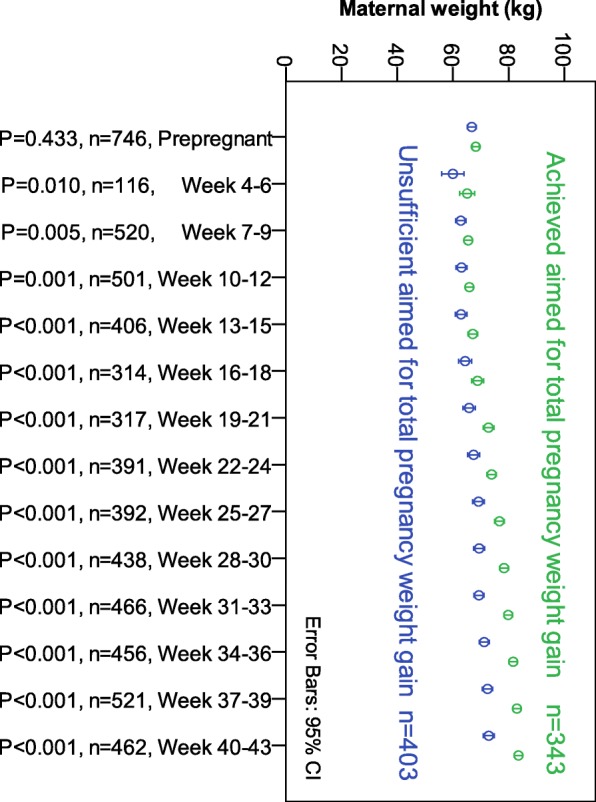
Weight measurements for 746 women with hyperemesis gravidarum categorized as achieving versus not achieving aimed for total weight gain (according to International Organization of Medicine recommendations per prepregnancy body mass index category [18]). Comparisons by Mann-Whitney test

**Fig. 3:**
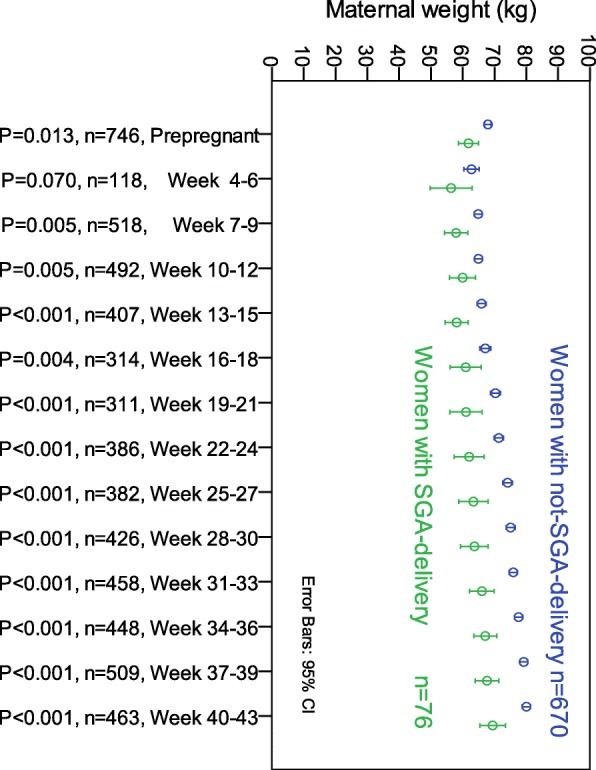
Maternal weights for 3-week interval during pregnancy for 746 women with hyperemesis gravidarum, classified according to pregnancy outcome. Small-for-gestational age (SGA) was defined by birth weight less than the 10th percentile for the actual gestational age at birth and sex, using sex- and gestational length-specific Norwegian neonatal weight charts [20]. Comparisons by Mann-Whitney test

The obstetric characteristics collected were gravidity, deliveries and any previous hyperemesis pregnancies. Smoking status at first admission was retrieved from patient files. Prepregnancy BMI was calculated from antenatal weight and height and classified according to the 2009 guidelines from the Institute of Medicine (IOM) [18]: underweight was defined as < 18.5 kg/m^2^, normal weight as 18.5 kg/m^2^–24.9 kg/m^2^, overweight as BMI 25.0 kg/m^2^–29.9 kg/m^2^ and obesity as > 30 kg/m^2^. Not achieving the minimal total weight gain goal was defined as a total pregnancy weight gain less than the minimum for the respective BMI category according to recommendations in the IOM guidelines [18]: 12.5–18.0 kg for women who were classified as underweight, 11.5–16.0 kg for women who were classified as normal weight, 7.0–11.5 kg for women who were classified as overweight and 5.0–9.0 kg for women who were classified as obese. Foetal ultrasound [19] was used to determine the gestational week for each maternal weight measurement. For each three-week interval (weeks 4–6, 7–9, 10–12 and so forth), the highest measured weight (either from inpatient or outpatient records) for each woman was noted. In Norway, for healthy pregnant women, weight measurements are not routinely performed this often during the 1st or 2nd trimester; therefore, we did not expect to have data for all women for all intervals. We have displayed the number of women with actual weight measurements for each three-week interval in Fig. 1, Fig. 2 and Fig. 3.

Initial pregnancy weight loss (as a percentage of body weight or absolute in kilos) at first admission and weight loss at nadir (the lowest of the registered weights during hospital admission) were computed from, respectively, self-reported prepregnancy weight until weight measured at first hospital admission or lowest weight measured during hospital stay. Inadequate early pregnancy weight gain was defined as weight < prepregnancy weight noted during the 13–15-week or 16–18-week interval, partly in line with gestational weight gain standards based on healthy women enrolled in the INTERGROWTH 21st project [21]. In our analyses, women who reached their prepregnancy weight by week 13–15 were marked as “regained” even if they were not weighed in the 16–18-week interval. Women who did not reach their prepregnancy weight by week 13–15 and did not have any new data by week 18 were marked as missing. Small-for-gestational age (SGA) was defined by birth weight less than the 10th percentile and large-for-gestational age (LGA) as larger than the 90th percentile for the actual gestational age at birth and sex, using sex- and gestational length-specific Norwegian neonatal weight charts [20].

Categorical variables were compared using Chi-square or Fisher’s test. Continuous variables are presented as the mean or median with 95% confidence intervals. Differences in weights between pregnancies with either inadequate or adequate weight gain or SGA/non-SGA outcomes were tested by the Mann-Whitney test. Differences among the four weight categories were tested by the Kruskal-Wallis test. Logistic regression was used to test which obstetrical/clinical or weight factors predicted inadequate weight gain or SGA outcome. Factors significant in univariate analysis were included in the final multivariate model. Smoking status is established as a major risk factor for poor growth/SGA [22] and was therefore retained in the model irrespective of initial univariate significance. Age and BMI were also considered important epidemiologic variables to merit inclusion in the logistic regression model. As weight measurements per 3-week interval were particularly incomplete (leaving 370 women with complete data to be included in the logistic regression), we compared baseline characteristics between those with complete versus incomplete data.

All tests were two-sided, and *p*-values < 0.05 were considered statistically significant.

Calculations were performed in SPSS (version 25, IBM corporation, Armonk, New York, USA).

This retrospective hospital cohort study was performed after approval from the hospital data protection officer at Haukeland University Hospital (Personvernombudet) for 2012/8379 (2002–2011 cohort) and 2017/1383 (2012–2016 cohort). The Regional Ethical Committee confirmed that individual patient consent could be waived (2018/2305) when the patients were classified as part of an internal control/quality study. All data have been anonymized and are reported according to the STROBE guidelines [23].

## Results

Out of 892 patients in the cohort, 888 could be categorized into four prepregnancy BMI groups, and for 784 women, the pregnancy lasted for > 24 weeks. In total, 746 of these pregnancies were singleton pregnancies in which the infant was classified as SGA/not SGA and LGA/not LGA. Baseline data for the 892 patients included in the study are shown in Table 1. Comparing these baseline data to the general Norwegian delivery population using the publicly available statistical bank from the Norwegian Birth Registry [24], we found no statistically significant differences regarding maternal age, parity or BMI (data not shown). However, through 2002–2016, 13.2% of 747,161 Norwegian women were smokers, a significantly higher prevalence than the 5.1% of the 837 hyperemesis patients in our cohort (*p* < 0.001).

**Table 1:** Baseline data for 892 women hospitalized with hyperemesis gravidarum at Haukeland University Hospital during 2002–2016

Variable	Mean	95% CI^a^	Median	95% CI
Age at admission (years)	28.1	27.8–28.5	28.0	28.0–29.0
Prepregnancy BMI^b,c^ (kg/m^2^)	24.4	24.1–24.7	23.6	23.3–23.9
Weight at admission^d^ (kg)	63.2	62.3–64.1	61.0	60.0–62.5
Weight loss at admission^e^ (kg)	4.4	4.2–4.6	4.0	4.0–4.0
Gestational age at admission^f^ (weeks)	9.4	9.2–9.6	8.6	8.4–9.0
	Number		Percentage	
Hyperemesis previously^g^				
HG in previous pregnancy	263		47.9	
No HG in previous pregnancy	286		52.1	
Smoking^h^				
Smoker	44		5.2	
Non smoker	795		94.8	
Gravidity				
Gravida 1	279		31.3	
Gravida > 2	613		68.7	
Parity				
Para 0	379		42.5	
Para > 1	513		57.5	
BMI categories^i,j^				
Underweight (< 20 kg/m^2^)	42		4.7	
Normal weight (20.0–24.9 kg/m^2^)	514		57.9	
Overweight (25.0–29.9 kg/m^2^)	230		25.9	
Obese (> 30 kg/m^2^)	102		11.5	

^a^: Confidence Interval, ^b^: Body Mass Index, ^c^: *n* = 4 missing values, ^d^: *n* = 1 missing value, ^e^: Lowest weight at admission, *n* = 22 missing values, ^f^: Gestational Age, as assessed by ultrasound measurement [19], ^g^: *n* = 64 missing values out of 613 women with any earlier pregnancy (Gravida > 2), ^h^: *n* = 53 missing values, ^i^: Body Mass Index, categorized according to Institute of Medicine (IOM) 2009 guidelines [18], *j*: *n* = 4 missing values

At first admission, women classified as underweight had lost less absolute weight (kg) than those in the other BMI groups, but neither percentage weight loss nor gestational age differed among weight groups (Supplementary Table 1). Despite a larger proportion of underweight women who regained their prepregnancy weight at 15 or 18 weeks (77% compared to 51% for the other weight groups), a smaller proportion of these women achieved their minimal total weight gain goal (47% compared to 54% for the other weight groups), and a larger proportion of these pregnancies ended with an SGA outcome (28% compared to 9%), all *p* < 0.041, Supplementary Table 1). Figure 1 illustrates the different weight gain patterns for the four prepregnancy weight categories; maternal weight persisted as significantly different for each of the measured three-week intervals throughout the entire pregnancy, all *p* < 0.001, Krúskal Wallis. Women not achieving their BMI-specific total pregnancy weight gain goal had a significantly larger weight loss at hospital admission, and a significantly higher proportion of these women did not regain their prepregnancy weight at 13–18 weeks of gestation and delivered an infant born SGA (all *p* < 0.001, Table 2).

**Table 2:** Characteristics for 746 women hospitalized due to hyperemesis gravidarum at Haukeland university Hospital during 2002–2016, comparing women with inadequate and adequate weight gain during pregnancy according to BMI-category^a^

Variable	Inadequate weight gain according to BMI-category^b^ (*n* = 343)		Adequate weight gain according to BMI-category (*n* = 403)		*P*-value Man-Whitney test
	Median	95% CI^c^	Median	95% CI	
Age at admission (years)	28.0	28.0–29.0	28.0	27.0–29.0	0.931
Weight loss at admission ^d^ (kg)	5.0	4.0–5.0	4.0	3.0–4.0	< 0.001
Weight loss at admission^e^ (%)	7.3	6.7–7.5	5.4	5.0–5.8	< 0.001
Gestational age at admission^f^ (weeks)	9.0	8.4–9.3	8.6	8.4–9.1	0.379
Body mass index (kg/m^2^)	23.6	23.1–23.9	23.9	23.2–24.7	0.433
	Number	%	Number	%	*P*-value Chi-Square test
Parity					0.720
Nulliparous	136	39.7	165	40.9	
Multiparous	207	60.3	238	59.1	
Hyperemesis in previous pregnancy^g^					0.422
HG previously	105	47.5	110	43.8	
No HG previously	116	52.5	141	56.2	
Prepregnancy weight regained at 12–15 or 16–18 weeks^h^					< 0.001
Not regained	127	71.8	53	26.1	
Regained	50	28.2	150	73.9	
SGA (*n* = 708)					< 0.001
SGA^i^	54	16.7	16	4.2	
Not SGA	270	83.3	368	95.8	
LGA (*n* = 708)					< 0.001
LGA^j^	11	3.4	39	10.2	
Not LGA	313	96.6	345	89.8	

^a^: *n* = 146 missing values out of 892 patients in the study, ^b^: Body Mass Index, categorized according to Institute of Medicine (IOM) 2009 [18], ^c^: Confidence Interval, ^d^: *n* = 22 missing values, ^e^: *n* = 1 missing value,

^f^: Gestational Age, as assessed by ultrasound measurement [19], ^g^: *n* = 472 out of 526 women with any earlier pregnancy (Gravida > 2), *n* = 54 missing values, ^h^: Regained = regained prepregnancy weight by week 13, 14, 15, 16, 17 or 18, *n* = 366 missing values, ^i^: Small for Gestational age according to Norwegian sex- and gender adjusted weight charts [20], *n* = 38 missing values, ^j^: Large for Gestational age according to Norwegian sex- and gender adjusted weight charts [20], *n* = 38 missing values

Using logistic regression, we identified not regaining prepregnancy weight by week 13–18 (OR 7.05, 95% CI 4.24–11.71, p < 0.001) as the only independent significant factor of inadequate weight gain determined by BMI category, as shown in Table 3. All baseline factors described in Table 1 were tested as individual (univariate) risk factors for inadequate maternal weight gain (data not shown). Except for those retained in the final model, none were of statistical significance. We have complete data for all included variables for 370 of the 892 women. Comparisons of the baseline clinical factors for the 370 women included in the logistic model with those for the group with missing data (Supplementary Table 2) revealed no significant differences except for the ratio of instances of hyperemesis gravidarum in previous pregnancies, a factor not significant in the logistic regression (data not shown). As illustrated in Fig. 2, maternal weight measured at each three-week interval differed significantly between women achieving the total weight gain goal and those who did not beginning as early as week 4–6 (*p* = 0.010) and continued to remain significantly different throughout pregnancy (all *p* < 0.005, Mann-Whitney U-test).

**Table 3:** Logistic regression for 370 women treated for hyperemesis gravidarum, predicting inadequate total maternal weight gain^a^ (*n* = 175 women did not achieve aimed weight gain)

	Univariate OR^**b**^	95% CI^**c**^	***p***-value	Multivariate OR	95% CI	***p***-value
Weight loss prepregnancy to nadir^d^	1.17	.09–1.26	< 0.001	1.05	0.97–1.15	0.245
Prepregnancy weight regained week 13-18^e^						
Regained *n* = 193	1			1		
Not Regained *n* = 177	7.26	4.59–11.49	< 0.001	7.05	4.24–11.71	< 0.001
Smoking status						
Smoking *n* = 16	1			1		
Not smoking *n* = 354	0.86	0.31–2.36	0.772	0.72	0.23–2.24	0.568
Body mass index	1.01	0.97–1.05	0.701	0.96	0.91–1.01	0.094
Age at admission	0.99	0.96–1.03	0.772	1.01	0.96–1.05	0.775

^a^: As aimed due to prepregnancy Body Mass Index, categorized and using limits for aimed total maternal weight gain according to Institute of Medicine (IOM) 2009 guidelines [18], ^b^: Odds ratio, ^c^: Confidence interval,

^d^: Lowest registered weight before start of treatment, ^e^: Regained = Regained prepregnancy weight by week 13, 14, 15, 16, 17 or 18

Significant risk factors for delivering an infant born SGA included being nulliparous, having inadequate total weight gain, not having regained prepregnancy weight by week 13–18 and having a low prepregnancy BMI (univariate logistic regression, all *p* < 0.026). Smoking, documented as a general risk factor for growth restriction, was included in the analyses but was not found to be an independent risk factor in our hyperemesis cohort. Nulliparous status, prepregnancy BMI and inadequate total weight gain were independent predictors of SGA (all *p* < 0.028) according to multivariate logistic regression analysis. Not regaining prepregnancy weight by week 13–18 was still statistically significant even after adjusting for the other risk factors (OR 2.66 with 95% CI 1.11–6.34), with a *p*-value of 0.028 (Table 4). Similarly, as described for the total weight gain regression, we had 361 women with complete data for the SGA prediction. Compared with the group with incomplete data, these women only exhibited a significant difference in gestational age, showing a slightly lower gestational age (8.6 weeks compared to 9.0 weeks, *p* = 0.048 Mann-Whitney, Supplementary Table 3), a factor not significant in univariate analysis (data not shown).

**Table 4:** Logistic regression for 361 women treated for hyperemesis gravidarum, predicting SGA^a^ (*n* = 39 singletons were defined as SGA)

	Univariate OR^b^	95% CI^c^	*p*-value	Multivariate OR	95% CI	*p*-value
Smoking						
Not smoking *n* = 344	1			1		
Smoking n = 17	2.72	0.84–8.79	0.095	3.05	0.84–11.13	0.091
Parity						
Para > 1 *n* = 195	1			1		
Para 0 *n* = 166	3.39	1.63–7.05	0.001	4.12	1.69–10.08	0.002
Prepregnancy BMI^d^	0.90	0.83–0.99	0.026	0.88	0.80–0.98	0.017
Weight gain week 13-18^e^						
Regained *n* = 187	1			1		
Not regained *n* = 174	3.54	1.67–7.51	0.001	2.66	1.11–6.34	0.028
Minimum total weight gain^f^						
Achieved *n* = 189	1			1		
Not achieved *n* = 172	4.23	1.94–9.19	< 0.001	3.09	1.29–7.39	0.011
Age at admission	0.96	0.90–1.02	0.182	1.05	0.98–1.14	0.177

^a^: Small for Gestational age according to Norwegian sex- and gender adjusted weight charts [20],

^b^: Odds ratio, ^c^: Confidence interval, ^d^: Body mass index before pregnancy,

^e^: Regained = Regained prepregnancy weight by week 13, 14, 15, 16, 17 or 18,

^f^: Whether patients achieved minimal aimed weight gain specific for their category of BMI, categorized and using limits for aimed maternal weight gain according to Institute of Medicine (IOM) 2009 guidelines [18]

We also found that the incidence of the weight-dependent predictors varied significantly across the four prepregnancy BMI groups, as shown in Table 2. Figure 3 shows a highly statistically significant difference from early pregnancy (weeks 7–9) when comparing maternal weights measured for each 3-week interval, as women delivering an infant born SGA had significantly lower weight throughout pregnancy than those not delivering an infant born SGA (all *p*-values < 0.005; Mann-Whitney U-test). Similar significant differences were found when comparing weights for women delivering an infant born LGA (*n* = 52) versus those not delivering an infant born LGA (Supplementary figure).

## Discussion

We have shown that insufficient early maternal weight gain in hyperemesis pregnancies is an independent predictor for not achieving the minimum total maternal weight gain goal with an OR of 7.05, as well as an independent risk factor for SGA with an OR of 2.66.

Although insufficient total weight gain has been determined to be strongly correlated with foetal weight [5, 6], the effect of the timing of weight gain during pregnancy has been less studied, and the results are conflicting. Some studies have concluded that weight gain during the 2nd and 3rd trimester is most important regarding foetal outcome [25, 26], while others have found early weight gain to be more crucial [17, 27, 28].

Retnakaran et al. [17] performed a prospective study on 1164 healthy, Chinese and generally lean women in which they measured weight and height at a median of 19.9 weeks before pregnancy. In this study, weight gain until week 14 and during week 14–18, in which periods birth weight increased by 13.6 g/kg (95% CI 3.2–24.2) and 26.1 g/kg (95% CI 3.8–48.4), respectively, were independent predictors of infant birth weight. Weight change during the latter half of pregnancy was not an independent predictor of birth weight. These observations align with our results, in spite of the long time interval from the prepregnancy measurement to conception. Catov et al. [28] performed a prospective study on 651 dominantly overweight/obese women during first trimester and identified that large gestational weight gain until 20 weeks, regardless of the later rate of weight gain, increased the risk of an LGA outcome (OR 2.93, 95% CI 1.16–7.41), in line with our findings. These authors described a non-significant relation between low early gestational weight gain followed by high later gain and a reduced risk of SGA outcome (OR 0.55, 95% CI 0.29–1.07). Brown et al. [27] included 389 women preconception and identified prepregnancy BMI and first trimester and second trimester weight gain but not third trimester weight gain as independent predictors of infant birth weight. Their study found that women with first trimester weight loss delivered babies with significantly lower birth weight than women with a first trimester weight gain above the median. However, their study was not sufficiently powered to investigate weight gain per BMI categories.

We have not identified any publications specifically investigating 1st trimester maternal weight gain as a predictor of adverse outcomes in hyperemesis pregnancies. The Norwegian mother and child cohort study investigating adverse outcomes in HG pregnancies [29] does not report weight data during 1st trimester and did not find any increased OR for SGA or other adverse pregnancy outcomes when adjusting for total maternal weight gain. Dodds et al. reported that HG patients with < 7 kg total maternal weight gain during pregnancy had an increased risk of SGA outcome (OR 1.5, 95% CI 1.0–2.2) [5]. Stokke et al. [6] confirmed that women with total weight gain < 7 kg delivered significantly more SGA infants with a multivariate OR of 3.68 (95% CI 1.89–7.18). Neither Stokke’s nor Dodds’ studies provided BMI-specific cut-offs for the maternal weight gain goal. Our study provides this new information for a large cohort of women treated for HG.

In our study, a higher percentage of underweight patients regained their prepregnancy weight by weeks 13–18 (77.3%) than that of the other BMI groups, especially the obese group, where only 32.6% achieved their prepregnancy weight by this timepoint. These differences might be explained by the amount of weight lost initially. As shown in Supplementary Table 1, absolute weight loss (kilos) at admission was significantly less in the underweight group than in the other groups (*p*-value of < 0.001). This observation corresponds to the lower amount of weight loss needed for an underweight woman to reach the threshold of > 5% of prepregnancy weight, commonly considered an indication for hospitalization [3]. A lower percentage of underweight women achieved their minimum total pregnancy weight gain goal (46.7%) than that of the overweight group (62.7%). Although there was no linear tendency in the percentage of women who achieved their minimal pregnancy weight gain goal across the BMI groups, as the normal weight group and the obese group had success rates of 50.9 and 52.9%, respectively, these differences between groups were significantly different (*p* = 0.041 Chi-square). Our data illustrate that weight gain patterns within pregnancy time intervals are different among different BMI categories and that these factors need to be incorporated into models elucidating pregnancy outcomes*.*

In the multivariate logistic regression investigating risk factors for inadequate total maternal weight gain according to BMI class, maximum weight loss from prepregnancy to admission had a univariate positive OR of 1.17 (high initial weight loss predicted risk of inadequate total weight gain), while in the multivariate analysis, this initial weight loss was not identified as a significant independent factor. Fejzo et al. investigated the characteristics of a population group of HG patients who experienced a weight loss > 15% of prepregnancy weight. This study did not find any significant difference in either prepregnancy BMI or adverse foetal outcome between those with extreme weight loss versus those with more moderate weight loss [7]. This conclusion is in line with our study; extreme weight loss might not be a negative predictor when adjusted for other factors, such as achieving adequate early or total weight gain.

The retrospective study design is challenging; weights were measured by several recorders (outpatient and inpatient measurements), and prepregnancy BMI was self-reported by the patients. Women tend to underreport their weight [30], potentially leading to an underestimation of initial weight loss and estimated weight gain needed to reach prepregnancy weight, which may attenuate rather than overestimate any effect of early pregnancy weight gain. Similarly, women in the uppermost BMI group (obese) tend to report weight more towards “normal”, e.g., lower weight [31]. As prepregnancy self-reported weight was used for BMI classification, any underestimation might attenuate the effect of weight differences.

Using weight per three-week interval was an effort to accommodate the INTERGROWTH approach [21], which uses measurement intervals of 5 weeks (+ 1). This 5-week interval approaches the 6-week interval of 13–18 weeks used in our study. However, women are not routinely weighed every 3 weeks throughout pregnancy. We used a conservative approach: women who did not reach their prepregnancy weight by week 13–15 and did not have any new data by week 18 were marked as “missing”; however, this conservative approach may have reduced the number of data points used and the power of the analysis. We acknowledge that the lack of complete data is a limitation, leaving 361 women with sufficient early and late weight data to identify early weight gain as an independent significant factor. However, comparisons between the group of women with missing data to the group with complete data showed that these groups were not significantly different regarding any of the factors included in the logistic regression (Supplementary Tables 2 and 3). We thus consider the main findings unlikely to be significantly altered with a more complete dataset.

Our cohort is a retrospective convenient sample of all patients treated during a 15-year period, as such a power calculation was not performed a priori. Performing post-hoc power calculation is not statistical meaningful. However, in spite of a limited number of patients with full data variables (*n* = 361) we identify not regaining weight during early pregnancy as an independent factor for SGA with OR of 2.66 with 95% CI 1.11–6.34 with a *p*-value of 0.028. With a larger/more complete dataset it is probable that the confidence intervals will become smaller, and as such factors not reaching statistical significance in our sample (e.g smoking) might attain this.

Haukeland University Hospital is a tertiary hospital for Hordaland County (10% of the Norwegian population) and demographically representative of the whole population [24]. The baseline data for our hyperemesis cohort is no different from the general Norwegian population except in relation to smoking. The latter is in line with a recent meta-analysis describing the risk of hyperemesis in smokers compared to non-smokers with an OR of 0.40 (95% CI: 0.24–0.56) [32]. Our 15-year hospital cohort of 784 women with hyperemesis and weight data from early pregnancy is a fairly large study and should be considered representative for Norway/Scandinavian countries.

To the best of our knowledge, this is the first study to investigate early maternal weight development in HG pregnancies as a predictor for SGA outcome and inadequate total weight gain, adjusting for prepregnancy BMI and using maternal weight gain cut-offs specific for BMI groups. Our findings that insufficient early pregnancy weight gain impacts the risk of poor pregnancy outcome are highly clinically relevant. Hyperemesis patients should be provided medical therapy aiming at reducing nausea and nutritional therapy to promptly reverse their first-trimester weight loss.

## Conclusion

Inadequate total maternal weight gain during pregnancy, not regaining prepregnancy weight by week 13–18 and having a low prepregnancy BMI may be considered independent risk factors for delivering a baby that is small for gestational age in pregnancies complicated by hyperemesis gravidarum. Regaining prepregnancy weight by week 13–18 is also an independent predictor of achieving the minimum total maternal weight gain goal specific for BMI classifications. Prepregnancy BMI and early pregnancy weight gain in HG pregnancies matters for pregnancy outcomes, highlighting that individualized nutritional treatment acknowledging differences according to prepregnancy BMI and severity of weight loss is important during this period.

## Supplementary information

**Additional file 1: Figure S1.** Maternal weights for 3-week interval during pregnancy for 746 women with hyperemesis gravidarum, classified according to pregnancy outcome. Large-for-gestational age (LGA) was defined by birth weight larger than the 90th percentile for the actual gestational age at birth and sex, using sex- and gestational length-specific Norwegian neonatal weight charts [20]. Comparisons by Mann-Whitney test.

**Additional file 2: Table S1.** Characteristics for 892 women hospitalized du to hyperemesis gravidarum at Haukeland university Hospital during 2002–2016, categorized by BMI^a^.

**Additional file 3: Table S2.** Comparison of baseline data between groups of patients included and excluded from logistic regression, predicting inadequate maternal weight gain^a^.

**Additional file 4: Table S3.** Comparing baseline data between groups of patients included and excluded from logistic regression, predicting SGA-infants^a^.

## Data Availability

Anonymized, limited versions of the datasets used and analysed during the current study are available from the corresponding author on reasonable request.

## Abbreviations

BMI: Body mass index
CI: Confidence interval
HG: Hyperemesis gravidarum
LGA: Large for gestational age
NVP: Nausea and vomiting in pregnancy
OR: Odds ratio
SGA: Small-for-gestational age
