# Correction to: Early maternal weight gain as a risk factor for SGA in pregnancies with hyperemesis gravidarum: a 15-year hospital cohort study

**DOI:** 10.1186/s12884-020-03020-9

**Published:** 2020-05-29

**Authors:** Tale Meinich, Jone Trovik

**Affiliations:** 1 Department of Clinical Science, University of Bergen, Jonas Lies vei 72, 5053 Bergen, Norway; 2 Department of Obstetrics and Gynaecology, Haukeland University Hospital, Jonas Lies vei 72, 5053 Bergen, Norway

**Correction to: BMC Pregnancy and Childbirth (2020) 20:255**

**https://doi.org/10.1186/s12884-020-02947-3**

Following publication of the original article [1], the authors identified an error in Fig. 1. The correct figure is given below.

**Fig. 1:**
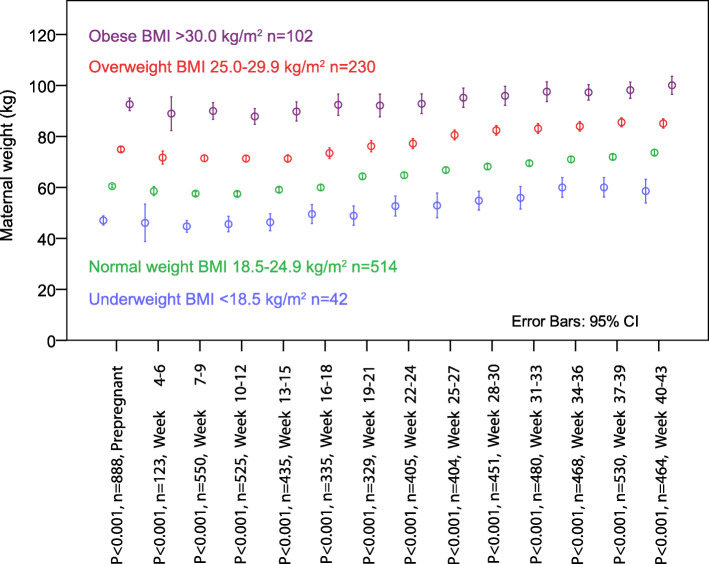
Weight gain patterns for 888 women hospitalized for hyperemesis gravidarum according to their prepregnancy body mass index (BMI) categories. Weight measured during 3-weeks interval during pregnancy until delivery. Comparisons by Kruskal-Wallis test

The original article has been corrected.
