# Supplementary figures and images for: Early maternal weight gain as a risk factor for SGA in pregnancies with hyperemesis gravidarum: a 15-year hospital cohort study
Source: BMC Pregnancy Childbirth. 2020 Apr 28;20:255. doi: 10.1186/s12884-020-02947-3 (PMC7189646; doi:10.1186/s12884-020-02947-3)

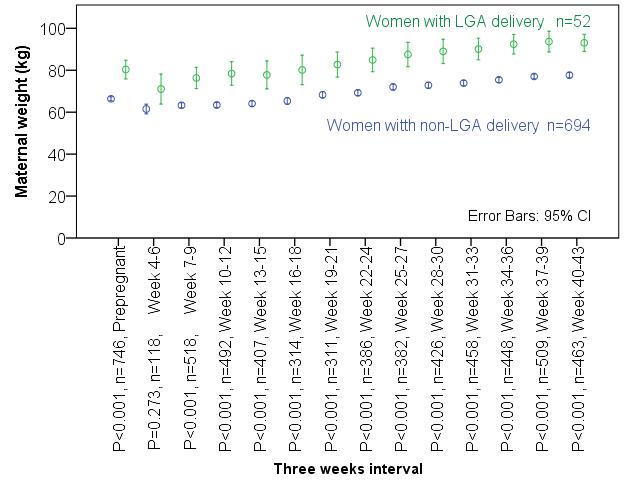

Supplement: Supplementary file 1 — Additional file 1: Figure S1. Maternal weights for 3-week interval during pregnancy for 746 women with hyperemesis gravidarum, classified according to pregnancy outcome. Large-for-gestational age (LGA) was defined by birth weight larger than the 90th percentile for the actual gestational age at birth and sex, using sex- and gestational length-specific Norwegian neonatal weight charts [20]. Comparisons by Mann-Whitney test. [file 12884_2020_2947_MOESM1_ESM.jpg]
