# Supplementary material for: Early maternal weight gain as a risk factor for SGA in pregnancies with hyperemesis gravidarum: a 15-year hospital cohort study
Source: BMC Pregnancy Childbirth. 2020 Apr 28;20:255. doi: 10.1186/s12884-020-02947-3 (PMC7189646; doi:10.1186/s12884-020-02947-3)
Supplement: Supplementary file 2 — Additional file 2: Table S1. Characteristics for 892 women hospitalized du to hyperemesis gravidarum at Haukeland university Hospital during 2002–2016, categorized by BMIa. [file 12884_2020_2947_MOESM2_ESM.docx]

**Supplementary table 1:** Characteristics for 892 women hospitalized du to hyperemesis gravidarum at Haukeland university Hospital during 2002-2016, categorized by BMI^a^.

| Variable | Underweight prepregnancy BMI^a^ | | Normal weight prepregnancy BMI^a^ | | Overweight prepregnancy BMI^a^ | | Obese prepregnancy BMI^a^ | | P-value  Kruskal Wallis test |
| --- | --- | --- | --- | --- | --- | --- | --- | --- | --- |
|  | Median | 95% CI^b^ | Median | 95% CI | Median | 95% CI | Median | 95% CI |  |
| Age at admission (years) | 27.0 | 25.0-28.0 | 28.0 | 27.0-28.0 | 29.0 | 28.0-30.0 | 29.0 | 28.0-31.0 | 0.011 |
| Weight loss at admission ^c^ (kg) | 3.0 | 2.0-3.5 | 4.0 | 4.0-4.0 | 4.75 | 4.0-5.0 | 5.0 | 4.0-6.5 | <0.001 |
| Weight loss at admission^d^ (%) | 5.3 | 3.9-7.3 | 6.4 | 6.0-6.8 | 6.0 | 5.4-6.9 | 5.6 | 4.2-6.7 | 0.055 |
| Gestational age at admission^e^ (weeks) | 8.3 | 7.6-10.0 | 8.6 | 8.3-9.1 | 8.6 | 8.4-9.1 | 9.3 | 8.3-10.1 | 0.205 |
|  | Number | % | Number | % | Number | % | Number | % | P-value  Chi-Square test |
| Parity  Para 0  Para >1 | 20  22 | 47.6  52.4 | 247  267 | 48.1  51.9 | 79  151 | 34.3  65.7 | 30  72 | 29.4  70.6 | <0.001 |
| Hyperemesis in previous pregnancy^f^  HG previously  No HG previously | 13  9 | 59.1  40.9 | 147  151 | 49.3  50.7 | 75  77 | 49.3  50.7 | 28  49 | 36.4  63.6 | 0.134 |
| Prepregnancy weight regained at 12-15 or 16-18 weeks^g^  Not regained  Regained | 5  17 | 22.7  77.3 | 107  133 | 44.6  55.4 | 49  45 | 52.1  47.9 | 31  15 | 67.4  32.6 | 0.003 |
| Minimal aimed for total weight gained during pregnancy^h^  Not achieved  Achieved | 16  14 | 53.3  46.7 | 215  223 | 49.1  50.9 | 72  121 | 37.3  62.7 | 40  45 | 47.1  52.9 | 0.041 |
| SGA^i^  Not SGA | 9  23 | 28.1  71.9 | 46  396 | 10.4  89.6 | 15  173 | 8.0  92.0 | 6  77 | 7.2  92.8 | 0.013^k^ |
| LGA^j^  Not LGA | 0  32 | 0  100.0 | 19  423 | 4.3  95.7 | 19  169 | 10.1  89.9 | 13  70 | 15.7  84.3 | <0.001^k^ |

a: Body Mass Index, categorized according to Institute of Medicine (IOM) 2009 ([18](applewebdata://6541B163-8873-4ADB-9FA3-B4B192EAE19B#_ENREF_18)), n=4 missing values for BMI

b: Confidence Interval, c: n=22 missing values, d: n=1 missing values, e: Gestational Age, as assessed by ultrasound measurement (19), f: Out of 613 women with any earlier pregnancy (Gravida >2), n=64 missing values, g: Regained = regained prepregnancy weight by week 13, 14, 15, 16, 17 or 18, n=489 missing values,

h: As determined per BMI category according to 2009 IOM-guidelines ([18](applewebdata://6541B163-8873-4ADB-9FA3-B4B192EAE19B#_ENREF_18)), n=146 missing values,

i: Small for Gestational age according to Norwegian sex- and gender adjusted weight charts ([21](applewebdata://6541B163-8873-4ADB-9FA3-B4B192EAE19B#_ENREF_21)), n=146 missing values, j: Large for Gestational age according to Norwegian sex- and gender adjusted weight charts ([21](applewebdata://6541B163-8873-4ADB-9FA3-B4B192EAE19B#_ENREF_21)), n=146 missing values, k: Fischer´s exact test
