# Supplementary material for: Early maternal weight gain as a risk factor for SGA in pregnancies with hyperemesis gravidarum: a 15-year hospital cohort study
Source: BMC Pregnancy Childbirth. 2020 Apr 28;20:255. doi: 10.1186/s12884-020-02947-3 (PMC7189646; doi:10.1186/s12884-020-02947-3)
Supplement: Supplementary file 3 — Additional file 3: Table S2. Comparison of baseline data between groups of patients included and excluded from logistic regression, predicting inadequate maternal weight gaina. [file 12884_2020_2947_MOESM3_ESM.docx]

**Supplementary table 2:** Comparison of baseline data between groups of patients included and excluded from logistic regression, predicting inadequate maternal weight gain^a^.

|  | Included patients (n=370) | | Excluded patients (n=522) | | P-value  Man-Whitney |
| --- | --- | --- | --- | --- | --- |
|  | Median | 95% CI^b^ | Median | 95% CI |  |
| Age at admission (years) | 29.0 | 28.0-29.0 | 28.0 | 27.0-28.0 | 0.051 |
| Weight at admission^c^ (kg) | 61.0 | 59.0-62.5 | 62.0 | 60.0-63.5 | 0.247 |
| Weight loss at admission^d^ (kg) | 4.0 | 4.0-4.0 | 4.0 | 4.0-4.5 | 0.608 |
| Prepregnancy BMI^e,f^ (kg/m^2^) | 23.3 | 22.8-23.9 | 23.8 | 23.4-24.2 | 0.268 |
| Gestational age at admission (weeks)^g^ | 8.9 | 8.4-9.1 | 8.6 | 8.3-9.0 | 0.067 |
|  | Number | Percentage | Number | Percentage | P-value  Chi-Square test |
| Hyperemesis previously^i^  HG in previous pregnancy  No HG in previous pregnancy | 93  127 | 42.3  57.7 | 170  159 | 51.7  48.3 | 0.031 |
| Smoking^j^  Smoker  Non smoker | 16  354 | 4.3  95.7 | 28  441 | 6.0  94.0 | 0.288 |
| Parity  Para 0  Para >1 | 170  200 | 45.9  54.1 | 209  313 | 40.0  60.0 | 0.079 |
| BMI categories ^k^  Underweight (<20 kg/m^2^)  Normal weight (20.0-24.9 kg/m^2^)  Overweight (25.0-29.9 kg/m^2^)  Obese (>30 kg/m^2^) | 19  223  88  40 | 5.1  60.3  23.8  10.8 | 23  291  142  62 | 4.4  56.2  27.4  12.0 | 0.533 |

a: As aimed due to prepregnant Body Mass Index, categorized and using limits for aimed total maternal weight gain according to Institute of Medicine (IOM) 2009 guidelines ([18](applewebdata://7E4D2335-7284-40F0-A598-47E3071FAB9C#_ENREF_18)), b: Confidence Interval, c: n=1 missing value,

d: n=22 missing values, e: n=4 missing values, f: Body Mass Index, categorized according to Institute of Medicine (IOM) 2009 ([18](applewebdata://C4F6A279-DAB6-4BF6-85A1-205A91670641#_ENREF_18)), g. Gestational Age, as assessed by ultrasound measurement (19), h: n=146 missing values, i: Out of 613 women with any earlier pregnancy (Gravida >2), n=64 missing values, j: n=4 missing values,

k: Body Mass Index, categorized according to Institute of Medicine (IOM) 2009 guidelines ([18](applewebdata://34D4BD38-390A-4688-821F-E22408D47DFC#_ENREF_18)), n=4 missing values
