# Supplementary material for: Early maternal weight gain as a risk factor for SGA in pregnancies with hyperemesis gravidarum: a 15-year hospital cohort study
Source: BMC Pregnancy Childbirth. 2020 Apr 28;20:255. doi: 10.1186/s12884-020-02947-3 (PMC7189646; doi:10.1186/s12884-020-02947-3)
Supplement: Supplementary file 4 — Additional file 4: Table S3. Comparing baseline data between groups of patients included and excluded from logistic regression, predicting SGA-infantsa. [file 12884_2020_2947_MOESM4_ESM.docx]

**Supplementary table 3:** Comparing baseline data between groups of patients included and excluded from logistic regression, predicting SGA-infants^a^.

|  | Included patients (n=361) | | Excluded patients  (n=531) | | P-value  Mann-Whitney test |
| --- | --- | --- | --- | --- | --- |
|  | Median | 95% CI^b^ | Median | 95% CI |  |
| Age at admission (years) | 28.0 | 28.0-29.0 | 28.0 | 27.0-29.0 | 0.282 |
| Weight at admission^c^ (kg) | 61.0 | 59.0-62.0 | 62.0 | 60.0-63.5 | 0.203 |
| Weight loss at admission^d^ (kg) | 4.0 | 4.0-4.0 | 4.0 | 4.0-4.3 | 0.683 |
| Prepregnancy BMI^e, f^ (kg/m^2^) | 23.2 | 22.7-23.8 | 23.9 | 23.4-24.3 | 0.112 |
| Gestational age at admission^g^ (weeks) | 9.0 | 8.4-9.1 | 8.6 | 8.3-9.0 | 0.048 |
|  | Number | Percentage | Number | Percentage | P-value  Chi-Square test |
| Maternal weight gain according to BMI-group^h^  Adequate  Inadequate | 189  172 | 52.4  47.6 | 214  171 | 55.6  44.4 | 0.376 |
| Hyperemesis previously^i^  HG in previous pregnancy  No HG in previous pregnancy | 95  118 | 44.6  55.4 | 168  168 | 50.0  50.0 | 0.604 |
| Smoking^j^  Smoker  Non smoker | 17  344 | 4.7  95.3 | 27  451 | 5.6  94.4 | 0.546 |
| Gravidity  Gravida 1  Gravida >2 | 128  233 | 35.5  64.5 | 151  380 | 28.4  71.6 | 0.026 |
| Parity  Para 0  Para >1 | 166  195 | 46.0  54.0 | 213  318 | 40.1  59.9 | 0.082 |
| BMI categories^k^  Underweight (<20 kg/m^2^)  Normal weight (20.0-24.9 kg/m^2^)  Overweight (25.0-29.9 kg/m^2^)  Obese (>30 kg/m^2^) | 19  222  82  38 | 5.3  61.5  22.7  10.5 | 23  292  148  64 | 4.4  55.4  28.1  12.1 | 0.203 |
